# NOD2 Supports Crypt Survival and Epithelial Regeneration after Radiation-Induced Injury

**DOI:** 10.3390/ijms20174297

**Published:** 2019-09-02

**Authors:** Chansu Lee, Changhoon Choi, Ho Suk Kang, Sung-Won Shin, Shin-Yeong Kim, Hee Chul Park, Sung Noh Hong

**Affiliations:** 1 Department of Medicine, Samsung Medical Center, Sungkyunwan University School of Medicine, Seoul 06351, Korea; 2 Department of Radiation Oncology, Samsung Medical Center, Seoul 06351, Korea; 3 Department of Internal Medicine, Hallym University Medical Center, Anyang-si, Gyeonggi-do 14068, Korea; 4 Department of Radiation Oncology, Sungkyunwan University School of Medicine, Seoul 06351, Korea

**Keywords:** NOD2, radiation, crypt survival, epithelial regeneration, organoid

## Abstract

Nucleotide-binding oligomerization domain-containing protein 2 (NOD2) affords stem cell protection and links microbes to intestinal epithelial regeneration. We investigated whether NOD2 status is associated with crypt survival and intestinal epithelial regeneration independent of microbiota-derived molecules. To assess crypt survival, a clonogenic microcolony assay was performed with 15 Gy of X-ray irradiation. The fractional crypt survival rate (46.0 ± 15.5% vs. 24.7 ± 9.2%, *p* < 0.01) and fractional EdU-positive crypt survival rate (29.8 ± 14.5% vs. 9.79 ± 4.37%, *p* = 0.015) were significantly decreased in the NOD2^−/−^ mice compared with the wild-type (WT) mice at 3.5 days after irradiation. To evaluate intestinal epithelial regeneration capability, organoid reconstitution assays were performed. Small bowel crypts of the WT and NOD2^−/−^ mice were isolated and seeded into Matrigel for 3D culture. In the organoid reconstitution assays, the number of organoids formed did not differ between the NOD2^−/−^ and WT mice. Organoid formation ability was also assessed after exposure to 5 Gy irradiation. Organoid formation ability was significantly decreased in the NOD2^−/−^ mice compared with the WT ones after exposure to 5 Gy irradiation (33.2 ± 5.9 vs. 19.7 ± 8.8/well, *p* < 0.01). NOD2 supports crypt survival after potentially lethal irradiation damage and is associated with intestinal epithelial regeneration.

## 1. Introduction

Crohn’s disease (CD) is a chronic, disabling disease of the gastrointestinal tract and its pathogenesis is still not completely understood. Nonetheless, it is thought that an abnormal mucosal immune response is elicited toward the luminal microbiota in genetically predisposed persons [1]. Among the identified genetic alterations, nucleotide-binding oligomerization domain 2 (NOD2) was the first to be observed and is the most influential mutation of CD. People who have NOD2 homozygous mutations are estimated to have roughly a 20-fold increased risk of CD [2]. NOD2 is an intracellular pattern recognition receptor that recognizes molecules containing a specific structure called muramyl dipeptide (MDP), a peptidoglycan constituent of both gram-positive and gram-negative bacteria. MDP-sensing NOD2 activates Mitogen-activated protein (MAP) kinase and the NF-kB pathway, which induces an inflammatory response [3]. NOD2 mutations in human cells lead to decreased NF-kB activation and reduced expression of pro-inflammatory cytokines, such as interleukin (IL)-8, IL-1β, and tumor necrosis factor-alpha (TNF-α) in response to MDP [4]. This may seem counterintuitive, as CD is generally associated with an increased expression of pro-inflammatory cytokines. 

Therefore, several hypotheses for NOD2-related pathogenesis have been proposed, including reduced expression and secretion of antimicrobial peptides [5,6], induced dysbiosis [7], and impaired autophagy through alterations in the recruitment of ATG16L1 to the basolateral membrane [8], leading to a hampered immune response. NOD2 is known to be expressed only in small bowel Paneth cells [9], but recent research has reported that NOD2 is highly expressed in intestinal stem cells (ISCs) [10]. Lgr5+ ISCs constitutively express the cytosolic innate immune sensor NOD2 at levels much higher than those in Paneth cells. In addition, MDP-stimulated NOD2 triggers stem cell survival, which leads to strong cytoprotection against oxidative stress-mediated cell death, indicating that NOD2 affords ISC protection and links microbes to intestinal epithelial regeneration [10]. However, whether NOD2 is associated with crypt survival and intestinal epithelial regeneration independent of microbiota-derived molecules is unclear. We investigated whether NOD2 status is associated with crypt survival and intestinal epithelial regeneration independent of microbiota-derived molecules.

## 2. Results

### 2.1. Dose-Dependent Radiation-Induced Intestinal Damage of NOD2^−/−^ Mice

**Figure 1:**
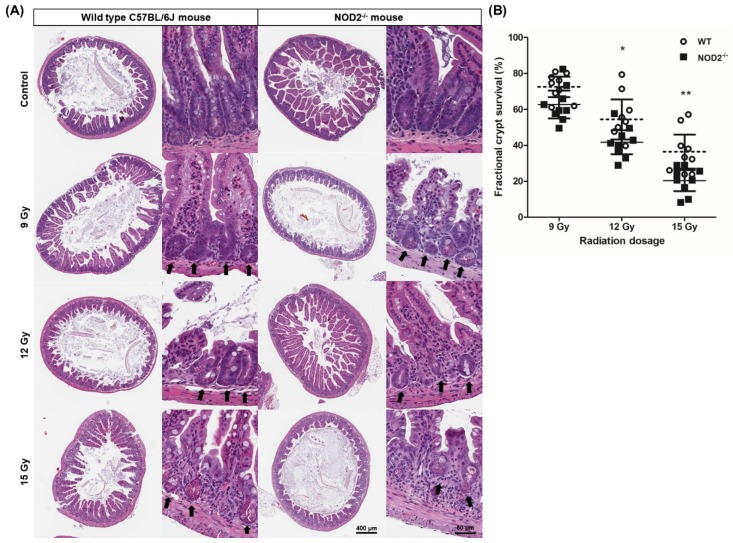
Dosage-dependent radiation damage of X-ray in the wild-type (WT) and NOD2^−/−^ mice. (**A**) Crypt microcolony assay. Arrows indicated the regenerative crypts. (**B**) Fractional crypt survival rate according to different doses of irradiation. Differences were evaluated by two-way ANOVA followed by Bonferroni post-test; * *p* < 0.05, and ** *p* < 0.01.

Because of the high sensitivity of ISCs in the small intestine to ionizing radiation and self-renewal capacity, the mouse small intestinal crypts were used as a standard model system for the assessment of in vivo crypt microcolony assay. To evaluate the susceptibility of radiation-induced damage and the ability of survival and regeneration in the small intestines of the NOD2^−/−^ mice (*n* = 3) compared with that of the wild-type (WT) mice (*n* = 3), crypt microcolony assays were performed using different doses of X-ray irradiation ranging from 9 to 15 Gy. The crypt-villus architecture and the number of crypts per circumference of the small intestine between the unirradiated small intestines of the WT and NOD2^−/−^ mice were identical in the Hematoxylin and Eosin (H&E)-stained histological sections (number of crypts/circumference of the intestine: 126.0 ± 14.7 vs. 121.3 ± 13.7, *p* > 0.05). As the irradiation doses increased, the crypt microcolony assays showed a dose-dependent reduction in the regenerated crypts of both WT and NOD2^−/−^ mice. In the NOD2^−/−^ mice, the destruction of crypt structure was more prominent than that in the WT mice (Figure 1A). The number of regenerative crypts and the fractional crypt survival rate were not significantly different in the condition of 9 Gy irradiation between both groups (number of crypts/circumference: 91.3 ± 9.3 vs. 76.1 ± 12.2, *p* > 0.05, fractional crypt survival rate: 72.5 ± 7.4% vs. 62.7 ± 10.0%, *p* > 0.05); however, these values decreased significantly in the NOD2^−/−^ mice compared with the WT mice with 12 Gy (number of crypts/circumference: 68.6 ± 18.2 vs. 50.6 ± 10.5, *p* < 0.05, fractional crypt survival rate: 72.5 ± 7.4% vs. 62.7 ± 10.0%, *p* > 0.05), and 15 Gy irradiation (number of crypts/circumference: 46.0 ± 15.5 vs. 24.7 ± 9.2, *p* < 0.01, fractional crypt survival rate: 46.0 ± 15.5% vs. 24.7 ± 9.2%, *p* < 0.01, Figure 1B). Crypt survival in the small intestine was suppressed more severely in the NOD2^−/−^ mice than in the WT mice.

### 2.2. Location-Dependent Crypt Survival after Radiation Injury

**Figure 2:**
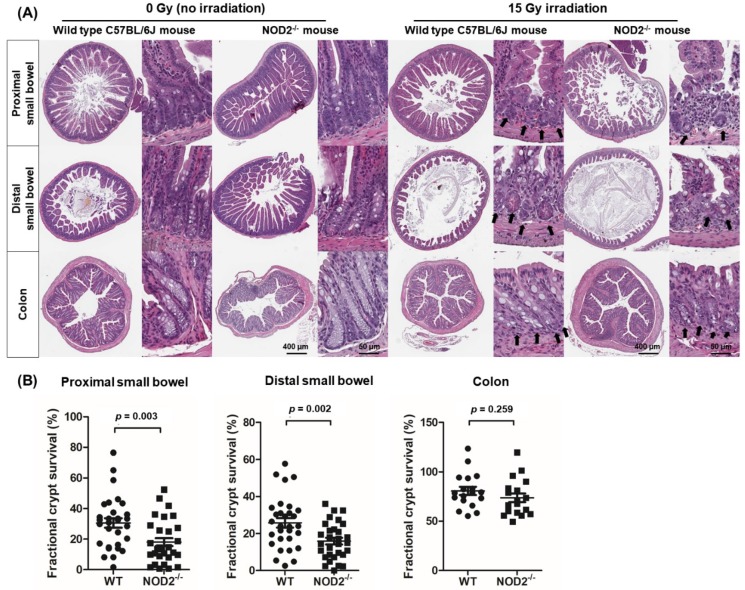
Location-dependent crypt survival after radiation injury in the WT and NOD2^−/−^ mice. (**A**) Crypt microcolony assay. Arrows indicated the regenerative crypts. (**B**) Fractional crypt survival rate according to different location of intestine. Differences were evaluated by two-way ANOVA followed by Bonferroni post-test.

Next, we compared crypt survival after radiation injury in different parts of the mouse intestine. The NOD2^−/−^ and WT mice were irradiated with 0 Gy (*n* = 6) and 15 Gy (*n* = 10) X-rays, and proximal and distal small intestine and colon samples were collected 3.5 days post-irradiation. The crypt numbers in the small intestine and colon tended to be diminished, showing different radiation sensitivities (Figure 2A). Quantitative analyses showed that the proximal and distal small intestines were more sensitive to radiation injury than the colon was, which is in accordance with a previous report that colonic epithelial stem cells are more radio resistant than their counterparts in the small intestine [11]. The fractional crypt survival rate of the proximal and distal small intestines of the NOD2^−/−^ mice was significantly diminished compared with that of the WT mice (proximal small intestine: 25.8 ± 15.9% vs. 14.0 ± 9.9%, *p* = 0.003, distal small intestine: 30.6 ± 18.1% vs. 17.1 ± 14.3%, *p* = 0.002), but not in the colon (80.7 ± 73.7 vs. 17.7 ± 18.8, *p* = 0.259. Figure 2B). These data indicate that radiation injury exerted a greater effect on small intestine crypt survival in the NOD2^−/−^ mice than in the WT mice.

### 2.3. EdU Assay of the Small Intestine after 15 Gy Irradiation

**Figure 3:**
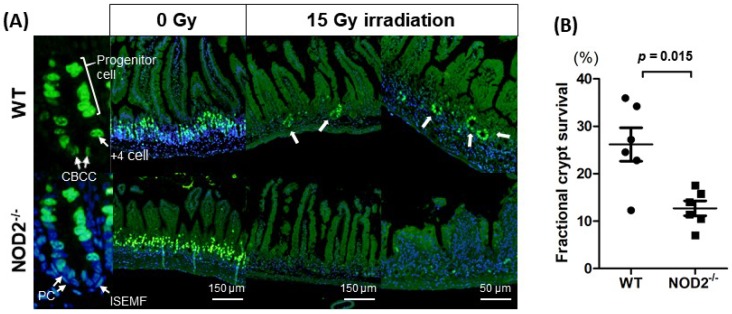
EdU assay in the WT and NOD2^−/−^ mice. (**A**) EdU assay of the small intestine after 15 Gy irradiation. Arrows indicated the surviving crypts. (**B**) Fractional EdU-positive crypt survival rate. Differences were evaluated by *t*-test. CBCC, crypt base columnar cell; PC, Paneth cells; ISEMF, intestinal subepithelial myelofibroblast.

Although counting surviving regenerative crypts in H&E-stained intestinal tissue sections is a standard in vivo crypt microcolony assay, predicting whether the crypts actually have the capacity to regenerate is difficult. The EdU was administrated intraperitoneally into the mice (*n* = 2) 2 h prior to euthanasia. Dividing S-phase cells are labeled by EdU and the EdU-positive cells in intestinal crypts are widely considered as a marker for ISC and progenitor cells [12]. The EdU-positive cells in intestinal crypts were clearly seen in the unirradiated control samples from the WT and NOD2^−/−^ mice, whereas the EdU-positive cells in crypts were markedly reduced in the X-ray-irradiated groups (Figure 3A). When a surviving crypt was defined as a crypt containing ≥5 EdU-positive cells, fractional crypt survival was significantly reduced in the NOD2^−/−^ mice compared with the WT mice (29.8 ± 14.5% vs. 9.79 ± 4.37%, *p* = 0.015). EdU staining may be useful in the precise visualization of regenerative crypts after irradiation. NOD2 depletion may be prone to damage of ISCs in the small intestine and could impair restoration of the regeneration ability.

### 2.4. Differentiation, Regeneration, and Apoptosis of Epithelial Cells Following Irradiation

**Figure 4:**
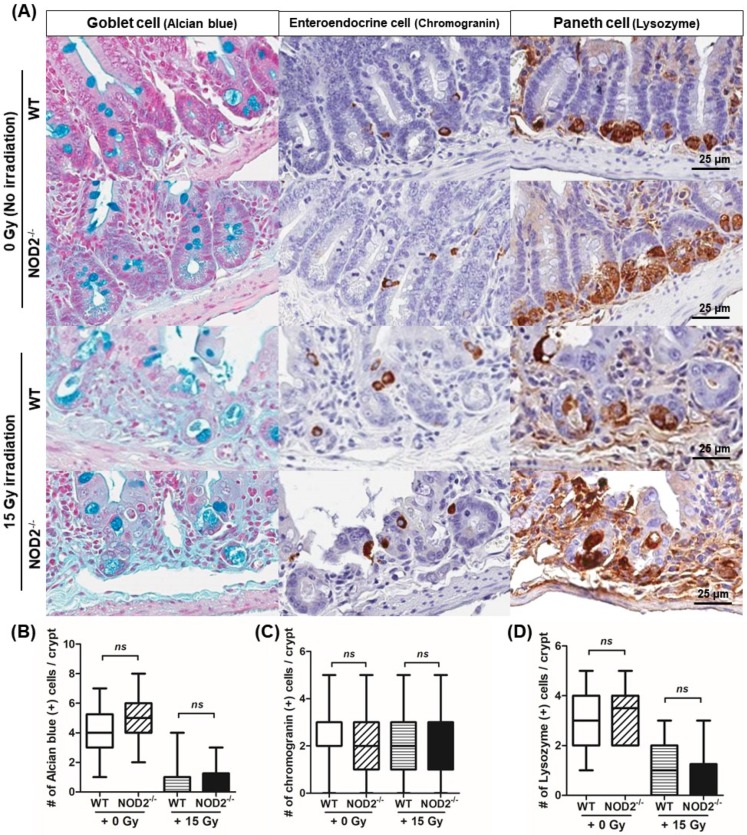
Immunohistochemical staining for goblet cells, enteroendocrine cells, and Paneth cells after 15 Gy irradiation in the WT and NOD2^−/−^ mice. (**A**) Immunohistochemical staining for goblet cells, enteroendocrine cells, and Paneth cells with alcian blue solution, chromogranin, and lysozyme antibody. (**B**) Alcian blue-stained goblet cells in the small intestinal crypt between the NOD2^−/−^ and WT mice, between the 0 and 15 Gy irradiations. (**C**) Chromogranin-positive enteroendocrine cells in the small intestinal crypt between the NOD2^−/−^ and WT mice, between the 0 and 15 Gy irradiations. (**D**) Lysozyme-positive Paneth cells in the small intestinal crypt between the NOD2^−/−^ and WT mice, between the 0 and 15 Gy irradiations.

To evaluate the differentiated cells including the goblet cells, enteroendocrine cells, and Paneth cells, alcian blue staining and Immunohistochemistry (IHC) was performed (Figure 4). The numbers of the goblet and enteroendocrine cells in the crypts were not significantly different between the NOD2^−/−^ and WT mice (4.3 ± 1.5/crypt vs. 4.9 ± 1.3/crypt and 2.3 ± 1.3/crypt vs. 2.2 ± 1.3/crypt) and between the 0 and 15 Gy irradiations (0.9 ± 1.0/crypt vs. 0.9 ± 0.9/crypt and 2.2 ± 1.5/crypt vs. 2.2 ± 1.3/crypt). In Paneth cells located on the crypt, the shape in irradiated samples was deformed, but the number was not different between the NOD2^−/−^ and WT mice (3.1 ± 1.0/crypt vs. 3.4 ± 1.1/crypt) and between the 0 and 15 Gy irradiations (1.1 ± 1.0/crypt vs. 1.0 ± 1.0/crypt).

**Figure 5:**
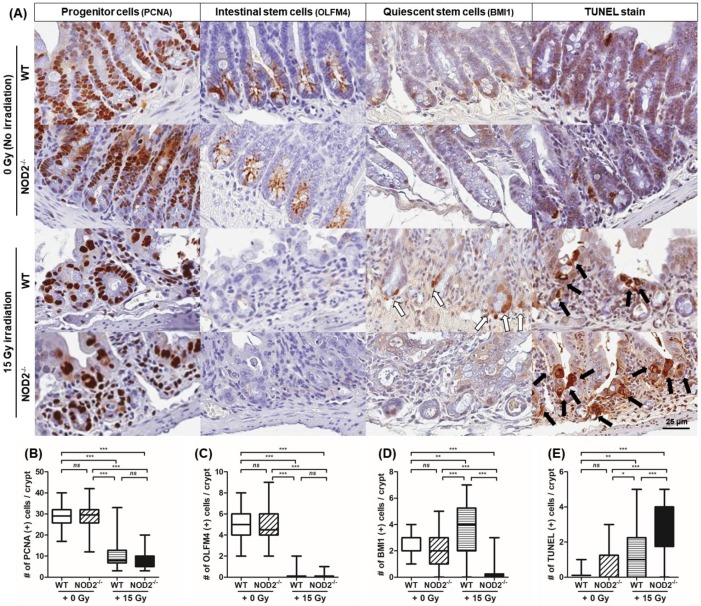
Immunohistochemical staining for progenitor cells, intestinal stem cells, and quiescent stem cells and TUNEL assay after 15 Gy irradiation in the WT and NOD2^−/−^ mice. (**A**) Immunohistochemical staining for progenitor cells, intestinal stem cells, and quiescent stem cells with OLFM4, PCNA, and BMI1. TUNEL stain for apoptic cells. White arrows indicated the BMI-positive cells. Black arrows indicated the TUNEL-positive cells. (**B**) PCNA-positive progenitor cells in the small intestinal crypt between the NOD2^−/−^ and WT mice and between the 0 and 15 Gy irradiations. (**C**) OLFM4-positive intestinal stem cells in the small intestinal crypt between the NOD2^−/−^ and WT mice and between the 0 and 15 Gy irradiations. (**D**) BMI1-positive quiescent stem cells in the small intestinal crypt between the NOD2^−/−^ and WT mice and between the 0 and 15 Gy irradiations. (**E**) TUNEL-positive apoptotic cells in the small intestinal crypt between the NOD2^−/−^ and WT mice and between the 0 and 15 Gy irradiations. Differences were evaluated in 10 crypts located on three different parts of the small bowel of three WT and NOD2^−/−^ mice by two-way ANOVA followed by Bonferroni post-test; * *p* < 0.05, ** *p* < 0.01, and *** *p* < 0.001.

To investigate the intestinal cells constituting intestinal crypts and their respective regeneration, IHC was performed (Figure 5A). Proliferating cell nuclear antigen (PCNA)-positive progenitor cells in the small intestinal crypt were not different between the unirradiated WT and NOD2^−/−^ mice (28.7 ± 4.9/crypt vs. 29.0 ± 6.2/crypt) and between the irradiated WT and NOD2^−/−^ mice (10.5 ± 6.7/crypt vs. 7.7 ± 3.7/crypt). However, the PCNA-positive progenitor cells in the unirradiated NOD2^−/−^ mice were significantly decreased compared with those in the irradiated WT (*p* < 0.001) and NOD2^−/−^ mice (*p* < 0.001, Figure 5B). Olfactomedin 4 (OLFM4)-positive ISCs in the small intestinal crypt were not different between the unirradiated WT and NOD2^−/−^ mice (4.8 ± 1.5 /crypt vs. 4.7 ± 1.7/crypt) and between the irradiated WT and NOD2^−/−^ mice (0.3 ± 0.4/crypt vs. 0.2 ± 0.3/crypt). However, the OLFM4-positive ISCs in the unirradiated NOD2^−/−^ mice were significantly decreased compared with those in the irradiated WT (*p* < 0.001) and NOD2^−/−^ mice (*p* < 0.001, Figure 5C).

B lymphoma Mo-MLV insertion region 1 homolog (BMI1)-positive quiescent stem cells were not different between the unirradiated WT and NOD2^−/−^ mice (2.4 ± 0.9/crypt vs. 2.2 ±1.3 /crypt), but there was a significant difference between the irradiated WT and NOD2^−/−^ mice (3.7 ± 2.1/crypt vs. 0.4 ± 0.9/crypt, *p* < 0.001). BMI-positive cells in the irradiated WT mice were significantly increased compared with those in the unirradiated mice (*p* < 0.001) and irradiated NOD2^−/−^ mice (*p* < 0.001). BMI-positive cells in the irradiated NOD2^−/−^ mice were significantly decreased compared with those in the unirradiated mice (*p* < 0.001) and irradiated NOD2*^−/−^* mice (*p* < 0.001). BMI1-positive cells in the small intestine of the irradiated NOD2^−/−^ mice were significantly decreased compared with those in the unirradiated WT and NOD2^−/−^ mice, as well as the irradiated WT mice (Figure 5D). Furthermore, Terminal deoxynucleotidyl transferase dUTP nick end labeling (TUNEL)-positive apoptotic cells within the crypts of the irradiated NOD2^−/−^ mice were significantly increased compared with those of the other mice (1.5 ± 1.4/crypt vs. 2.7 ± 1.5/crypt, *p* < 0.001), whereas TUNEL-positive apoptotic cells in the NOD2^−/−^ mice showed an increased tendency compared with those in the WT mice without statistical significance (0.2 ± 0.3 vs. 0.8 ± 1.0). TUNEL-positive cells in the irradiated NOD2^−/−^ mice were significantly increased compared with those in the unirradiated mice (*p* < 0.001) and irradiated WT mice (*p* < 0.001, Figure 4E). It has previously been noted that BMI1-positive cells are located on the +4 position and that they dramatically proliferate to clonally repopulate multiple contiguous crypts and villi after radiation-induced injury [13]. Thus, these data indicated that the increased susceptibility of radiation-induced injury in NOD2^−/−^ mice may be partially or completely attributed to the loss of BMI1-positive quiescent stem cells and the accumulation of TUNEL-positive apoptotic cells.

### 2.5. Organoid Reconstitution Assay

**Figure 6:**
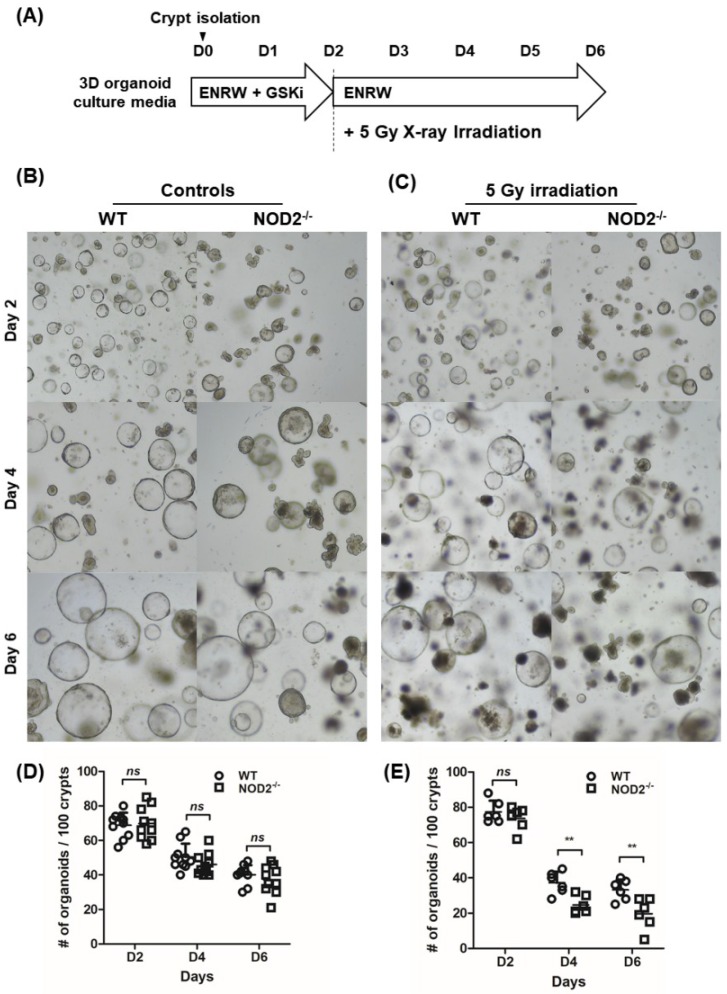
Organoid reconstitution assay in WT and NOD^−/−^ mice. (**A**) Organoid reconstitution assay protocol. Micrographs of non-treated (**B**) and 5 Gy X-ray-irradiated (**C**) organoids. The number of viable organoids per well in non-treated (**D**) and 5 Gy X-ray-irradiated (E) organoids. All differences were evaluated with two-way ANOVA followed by Bonferroni post-test; ** *p* < 0.01.

To evaluate intestinal epithelial regeneration capability, small bowel crypts of the WT and NOD2^−/−^ mice were cultured, and organoid reconstitution assays were performed (Figure 6A). The number of organoid formed was not decreased in the NOD^−/−^ mice compared with the WT mice (day 2: 68.8 ± 7.2/well vs. 69.8 ± 9.3/well, *p* > 0.05; day 4: 50.4 ± 7.7/well vs. 46.0 ± 6.4/well, *p* > 0.05; and day 6: 40.1 ± 5.5/well vs. 37.3 ± 8.3/well, *p* > 0.05; Figure 6B,E).

When organoids were irradiated with 5 Gy irradiation on day 2 (Figure 6C,F), organoid formation was significantly decreased in WT mice organoids on days 4 and 6 (day 4: no irradiation, 50.4 ± 7.7/well vs. 5 Gy irradiation, 37.2 ± 6.3 /well, *p* < 0.001 and day 6: 40.1 ± 5.5/well vs. 33.2 ± 5.9/well, *p* < 0.001, respectively). These results suggested that the epithelial regenerative ability of NOD2^−/−^ intestinal organoids is more susceptible to radiation injury.

## 3. Discussion

We investigated whether NOD2 status is associated with crypt survival and intestinal epithelial regeneration independent of microbiota-derived molecules. The number of surviving crypts and fractional crypt survival decreased significantly in the NOD^−/−^ mice compared with those in the WT mice. PCNA-positive progenitor cells and OLFM4-positive ISCs were not different in number between the irradiated WT and NOD2^−/−^ mice, whereas BMI1-positive quiescent stem cells were significantly decreased and TUNEL-positive apoptotic cells were significantly increased in the NOD2^−/−^ mice compared with the WT mice. In the organoid reconstitution assays, organoid formation did not differ between the NOD^−/−^ and WT mice, but the ability of organoid formation was decreased after exposure to 5 Gy irradiation.

Although NOD2 mutation is considered to be the most influential mutation for susceptibility to CD, the role of NOD2 in the pathogenesis of CD remains unclear. Currently, several hypotheses have been introduced; NOD2, a pattern recognition receptor, is expressed in Paneth cells, most of which are associated with the maintenance of homeostasis with intestinal microbiota through the modulation of antimicrobial peptides or autophagy [3]. Recent research has revealed that NOD2 expression is prominent in crypt base columnar cells, and NOD2 affords ISC protection [10]. In addition, NOD2 stimulation by MDP triggers stem cell survival, which leads to strong cytoprotection against oxidative stress-mediated cell death [10].

Although it is well described that Paneth cells support and nourish ISCs by providing niche signals, such as Wnt ligands (EGF, Wnt3, and the Notch ligand Dll4) [14], intestinal subepithelial myelofibroblasts (ISEMFs) and other components could play the same role. Nigro and colleagues reported that MDP released by intestinal microbiota could be a factor in protecting CBC cells, especially under stress conditions that are independent of Paneth cells [10]. Crypt proliferation in MDP-treated mice was increased starting 24 h after doxorubicin injection compared with the case of the nontreated mice, and complete restoration of the normal proliferation levels occurred in the MDP-treated mice 72 h post-doxorubicin injection. The capacity of the ISCs to regenerate crypts depended on their response to MDP through NOD2.

In this study, we applied radiation as a stressor and damager of ISCs. Without MDP stimulation, we demonstrated that NOD2-deficient ISCs were more severely damaged than normal ISCs were in vivo and in vitro. This means that NOD2 protects ISCs upon radiation-induced injury, and intestinal epithelial regeneration (or even restitution in the case of an injury) is clearly dependent on the presence of NOD2. In a resting state, intestinal homeostasis can be maintained by LGR5+ ISCs (stained with OFLM4 in this study). In conditions where LGR5+ ISCs are depleted, intestinal homeostasis can be maintained by the activation of LGR5-quiescent ISCs (stained with BMI1 in this study). LGR5-ISC populations are thought to play a similar role during intestinal regeneration following radiation-induced damage. BMI1-positive ISCs interconvert with more rapidly proliferating LGR5+ ISCs [15]. In this study, we demonstrated that BMI1-positive quiescent stem cells were decreased in the NOD2^−/−^ mice after irradiation. The BMI1+ cell population was increased after irradiation [16], and this study also demonstrated that the number of BMI1+ cells in crypts was increased in the WT mice. This result indicates that NOD2 supports not only LGR5+ ISCs but also BMI1+ cells.

Patients with CD may have an increased risk for severe acute radiation-related gastrointestinal complications [17]. Therefore, patients with CD are classified as a relative contraindication to radiation therapy. NOD2 mutations are strongly associated with the development of CD. We therefore selected ionizing radiation as a test damaging method in the NOD2^−/−^ mice, and our results may explain why CD patients are vulnerable to radiation therapy. To minimize the radiation injury to normal tissues around the target, intensity-modulated radiation therapy and 3D conformal radiotherapy should be considered [18].

**Figure 7:**
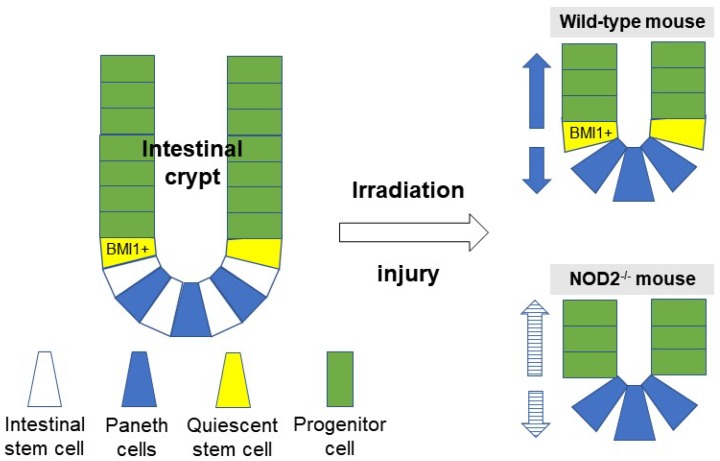
The proposed function of NOD2 after radiation-induced injury.

Our study has several limitations. First, whether the cytoprotection effect of NOD2 is a direct cell protection effect or whether it accelerates cell regeneration to maintain cell burden, or both, remains unclear. Second, we cannot exclude the role of other populations, such as immune cells, ISEMFs, and microbial components, in the regeneration process. Nevertheless, our study may give clues for understanding the association of NOD2 with CD. NOD2 mutations are likely to involve ISC dysfunction characterized by a delay in epithelial restitution, followed by intestinal injury that includes not only infection but also medication, and chemical or physical stress. NOD2 supports crypt survival through maintaining BMI1+ quiescent stem cells after potentially lethal irradiation damage and is associated with intestinal epithelial regeneration (Figure 7).

## 4. Materials and Methods

### 4.1. Animal Experiments

All animal experiments were conducted according to protocols approved by the Institutional Animal Care and Use Committees of the Samsung Biomedical Research Institute (SBRI) at Samsung Medical Center (approval number: 20150105005, approval date: 19 January 2015). The SBRI abides by the Institute of Laboratory Animal Resources guide and is also an accredited facility of the Association for Assessment and Accreditation of Laboratory Animal Care International. The experimental conditions and weight loss of the mice were monitored daily.

### 4.2. Mice and Irradiations

Wild-type (WT) C57BL/6J mice and NOD2^−/−^ mice with a C57BL/6 background (B6.129S1-Nod2tm1Flv/J, JAX#005763) were purchased from Jackson Laboratories (Bar Harbor, ME, USA) and housed under specific pathogen-free conditions. The mice were maintained in a 12 h light/12 h dark cycle and were fed ad libitum.

X-ray irradiation was performed with a Varian Clinac 6EX linear accelerator (Varian, Medical Systems, Palo Alto, CA, USA) at Samsung Medical Center, Seoul. The mice were anesthetized by intraperitoneal injection of 30 mg/kg zolazepam/tiletamine and 10 mg/kg xylazine prior to the irradiations. For X-ray irradiation, the mice were placed under a 2 cm thick water-equivalent bolus with a source-to-surface distance of 100 cm and a field size of 32 cm × 7 cm. The abdomen area, including the small and large intestines, was irradiated with graded single doses of 6 MV X-rays at a dose rate of 3.96 Gy per min.

### 4.3. Clonogenic Microcolony Assay

To compare the dose-dependent radiation damage of X-rays, the mice were exposed to 0 (no irradiation), 9, 12, and 15 Gy of X-ray irradiation and sacrificed after 84 h (3.5 days) [19]. Regenerative crypts referred to the atrophic or withering crypts with nuclear atypia. The number of surviving crypts per circumference of the transverse sectioned intestine was enumerated from hematoxylin and eosin (H&E)-stained sections. To assess location-dependent radiation damage from X-rays, the mice were exposed to 15 Gy of X-ray irradiation, and the samples were harvested from the proximal half of the small intestine (proximal small intestine), the distal half of the small intestine (distal small intestine), and the colon after 84 h [19]. Fractional crypt survival was defined as the percentage of the number of surviving crypts in the circumference of the intestine in irradiated mice to the number of crypts per cross-section from the same region of the intestine in unirradiated mice of the same strain and age [10].

### 4.4. EdU Assay

Two hours before sacrifice, 200 μg of 5-ethynyl-2′-deoxyuridine (EdU, Sigma-Aldrich, St. Louis, MO, USA) dissolved in phosphate buffered saline (PBS) was injected into the mice intraperitoneally. EdU incorporation into DNA was detected using the Click-iT™ EdU Alexa Fluor^®^ 488 imaging kit (Thermo Fisher Scientific, Waltham, MA, USA). A surviving crypt was defined as one containing ≥5 EdU-positive cells [20]. The number of surviving crypts per circumference of the small intestine was counted.

### 4.5. Immunohistochemistry (IHC)

After heat-induced epitope retrieval with citrate buffer, IHC was performed with chromogranin A (1:1000 dilution, Abcam, Cambridge, UK), lysozyme (1:3000 dilution, Abcam), OLFM4 (1:400 dilution, cell signaling technology, Danvers, MA, USA), PCNA (1:100 dilution, Abcam), and BMI1(1:400 dilution Abcam) antibodies. Alcian blue staining was also performed with alcian blue solution (Sigma-Aldrich) after epitope retrieval.

### 4.6. TUNEL Assay

Apoptosis-associated DNA fragmentation was detected by terminal deoxynucleotidyl transferase dUTP nick-end labeling (TUNEL) using an in situ cell death detection kit (Sigma-Aldrich). TUNEL was carried out according to the manufacturer’s instructions. Positive control sections were incubated with 10 U/mL recombinant DNase I solution, and a negative control was processed in the same manner with the omission of the terminal transferase enzyme.

### 4.7. Mouse Intestinal Crypt Isolation Culture

Murine small intestinal crypts were isolated as previously described [21]. Briefly, murine small intestinal crypts were isolated from six- to twelve-week-old WT and NOD2^−/−^ mice. The mice were euthanized, and the entire small intestine was harvested from the duodenum to the terminal ileum and flushed with ice-cold PBS. Each small intestine was cut into 5 mm fragments and then incubated in PBS containing 10 mM ethylenediaminetetraacetic acid (EDTA, Sigma, St. Louis, MO, USA) and 1 mM Dithiothreitol (DTT, Sigma) with gentle shaking at 4 °C for 30 min. The supernatant was removed, and the fragments were washed and vortexed with cold PBS. The washing solution was passed through a 70 μm pore cell strainer (BD Biosciences, Bedford, MA, USA), and the crypts were observed under a microscope. The isolated intestinal crypts were cultured in a 3D Matrigel matrix (BD Biosciences, Bedford, MA, USA) in advanced Dulbecco’s modified Eagle medium (DMEM)/Ham’s F-12 containing 1 × Antibiotic-Antimycotic, 2 mM Glutamax (Invitrogen, Waltham, MA, USA), 10 mM HEPES (Invitrogen), 1 × N2 (Invitrogen), 1 × B27 (Invitrogen), 50 ng/mL EGF, 1 mM *N*-acetylcysteine (Sigma), 100 ng/mL recombinant murine Noggin (Peprotech, Rocky Hill, NJ, USA), 500 ng/mL recombinant human R-spondin1 (Rspo1, R&D Systems, Minneapolis, MN, USA), and 100 ng/mL recombinant Wnt3a (R&D Systems). We added 2.5 μM GSK3 inhibitor (CHIR99021, Stemgent, Cambridge, MA, USA) for the first two days. The culture medium was replaced every 2 days.

### 4.8. Organoid Reconstitution Assay

Small intestinal crypts were plated at a concentration of 100 crypts per 25 µL of Matrigel into a 48-well plate. After the culture media were changed 2 days after plating, the intestinal organoids were exposed to 5 Gy of X-ray irradiation. Micrographs of the in vitro cultures were taken every day for 1 week by using a Leica SP2 MP-FLIM microscope (Leica Microsystems, Wetzlar, Germany). The total number of viable organoids per well was counted under a microscope [22].

### 4.9. Statistics

The statistical significance of the differences observed between experimental groups was analyzed using the statistical software GraphPad Prism v7.04 (GraphPad Software, La Jolla, CA, USA). Statistical significance was set at a level of *p* < 0.05.
